# Prevention of Postoperative Skin Disorders and Pressure Injuries in the Neurosurgical Park Bench Position Surgery: A Prospective Cohort Study

**DOI:** 10.7759/cureus.58552

**Published:** 2024-04-18

**Authors:** Kentaro Hara, Takahiro Uemura, Reika Tachibana, Ryosuke Kumashiro, Michiko Yamaguchi, Ichiro Kawahara, Masaki Fujioka

**Affiliations:** 1 Operation Center Division, National Hospital Organization Nagasaki Medical Center, Nagasaki, JPN; 2 Healthcare Management Research Center, Chiba University Hospital, Chiba, JPN; 3 Department of Anesthesiology, National Hospital Organization Nagasaki Medical Center, Nagasaki, JPN; 4 Department of Neurosurgery, Japan Community Health Care Organization Isahaya General Hospital, Nagasaki, JPN; 5 Department of Plastic and Reconstructive Surgery, National Hospital Organization Nagasaki Medical Center, Nagasaki, JPN

**Keywords:** surgical procedures, park bench positioning, neurosurgical, pressure injuries, postoperative cutaneous disorders

## Abstract

Background

In neurosurgical procedures where the park bench position is employed, the risk of perioperative pressure injuries is elevated due to the limited contact surface area, with the head and part of the upper torso extending beyond the surgical table. This study aimed to examine the effects of preventative measures against such injuries, proposing a potential standard for postural fixation in these surgeries.

Methods

Conducted at a medical center, from January 2017 to March 2023, this prospective cohort study involved participants aged 20 and above who underwent neurosurgical procedures in the park bench position under general anesthesia. The focus was on comparing the incidence of pressure injuries between intervention and control groups. The study adhered to the Strengthening the Reporting of Observational Studies in Epidemiology (STROBE) guidelines.

Results

Out of 65 patients enrolled, 28 were assigned to each of the intervention and control groups. The control group experienced 17 instances of postoperative pressure injuries and skin disorders in areas prone to pressure, such as the axillary and greater trochanter regions. Conversely, the intervention group reported no such incidents, underscoring the efficacy of meticulous surgical positioning and management of bodily pressure, temperature, humidity, and microclimate.

Conclusion

Implementing preventive measures in neurosurgical park bench procedures significantly reduces the incidence of postoperative pressure injuries and skin disorders. These findings advocate for the adoption of standardized postural fixation protocols in such surgeries, potentially influencing global clinical practices in neurosurgery.

## Introduction

A pressure injury occurs when pressure is applied to localized bony areas and skin, causing damage to the underlying tissues. This condition is worsened by shear forces [1]. In the operating theater, factors such as general anesthesia, chronic diseases, swelling, variations in body weight, blood loss over 800 ml, and surgeries lasting longer than five hours can lead to pressure injuries [2]. These injuries significantly affect patients by reducing their quality of life, increasing the risk of infections, and raising healthcare costs [3,4]. Therefore, preventing pressure injuries during surgery is a critical focus in perioperative nursing. This importance has grown as medical advancements have made longer and more complex surgeries possible [5].

The exigencies of surgical positioning, mandated by the need for an unimpeded operative field that ensures maneuverability, often necessitate robust fixation. Paradoxically, this can engender conditions incongruent with the imperatives of pressure injury prevention and management [6,7]. Previous reports have documented instances of pressure injury occurrence in the distinctive park bench position, a specialized lateral configuration employed in neurosurgical procedures [8]. Within this paradigm, where the cranium and a portion of the upper body extend beyond the confines of the operating table, the contact area is further diminished compared to the conventional lateral position, concomitantly heightening the proclivity for pressure injuries [9]. Moreover, in neurosurgical interventions, the inclination of the operating table towards the ventral side of the patient for optimal visualization of deep brain tissues augments the risk for pressure injuries, as rotational shear stress becomes more pronounced [10]. Augmenting the gamut of risk factors within the operating room, factors such as perspiration, surgeries surpassing six hours, hyperthermia, and elevated skin temperature have been correlated with the park bench position [10,11]. Remarkably, the incidence of pressure injuries in park bench position surgeries surpasses that reported for lateral decubitus configurations in the operating room [12-14]. Given the heightened susceptibility in park bench position surgeries, necessitating preemptive measures to avert pressure injuries becomes imperative.

This prospective observational study aimed to interrogate the repercussions of implementing preventative interventions against perioperative pressure injuries in the context of neurosurgical park bench positioning. We hypothesize that proactive measures to forestall a pressure injury can mitigate its occurrence even in park bench position surgeries.

## Materials and methods

Study design and ethical considerations

This single-center, prospective cohort study was conducted at the National Hospital Organization Nagasaki Medical Center, and was approved by the National Hospital Organization Medical Center Ethics Review Committee (no. 2022021). The study and its protocol were registered in the University Hospital Medical Information Network (UMIN) Clinical Trials Registry (UMIN000053254). The study was explained in detail to all the patients or their legal guardians, and written informed consent was obtained. The study design complied with the Strengthening the Reporting of Observational Studies in Epidemiology (STROBE) guidelines [15]. Statistical planning was done before accessing the data, and data analysis was performed after accessing the data [16].

Study setting and sampling

This study was conducted at a medical center between January 2017 and March 2023. All participants were 20 years of age or older at the time of neurosurgery park bench position surgery under general anesthesia. The exclusion criteria were as follows: children, emergency surgery patients, and patients with a history or family history of malignant hyperthermia. The sample size was calculated using G*Power 3.1 (Heinrich Heine University Düsseldorf, Germany) with an effect size of 0.5, power of 0.8, and alpha of 0.05, in reference to previous studies, with 28 cases in each group [17,18].

Outcome and data collection

The primary endpoint was the number of postoperative pressure injuries. The secondary endpoints were the number of postoperative skin disorders and the number of postoperative neurological disorders. Decisions were made for pressure ulcers according to the National Pressure Ulcer Advisory Panel (NPUAP) classification [19]. Data related to preoperative clinical findings (age, sex, height, weight, BMI, American Society of Anesthesiologists, or ASA, classification), preoperative physical findings (nutrition, paralysis, and edema), intraoperative factors (operating time, anesthesia time, blood loss, fluid volume, urine volume, mean body pressure during surgery, mean central temperature during surgery), and postoperative factors (postoperative pressure ulcers and skin disorders in the axilla and greater trochanter, postoperative pressure ulcers and skin disorders other than the axilla and greater trochanter, and postoperative neurological disorders) were collected from the study subjects. Postoperative reactive hyperemia was included if it persisted for more than 15 min, taking into account the time from the end of the operation to leaving the operating room.

Intraoperative pressure ulcer prevention

Intervention Group

The intervention group was included in this study between July 2019 and March 2023. Preoperative skin protection was achieved using the ESENTA Skin Capsule Spray® (#423288; ConvaTec, Tokyo, Japan) as a skin protectant on the lateral chest, greater trochanter, fibula head, and malleolus after entering the operating room in the lateral decubitus position. ALLEVYN Life® (#66801070; Smith & Nephew, Fukuoka, Japan) was then applied to the lateral chest, greater trochanter, fibula head, and malleolus.

**Figure 1:**
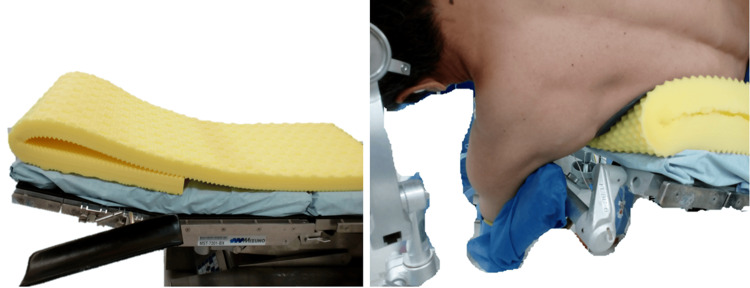
Installation of the operating table mattress and placement of the axillary area An additional mattress was used on the mattress attached to the operating table to remove the axillary pillow.

**Figure 2:**
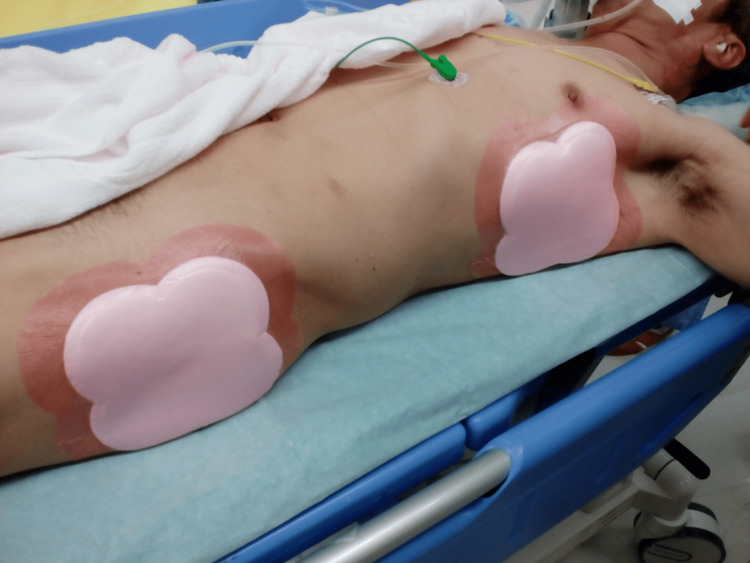
Affixing multilayered silicone foam mattresses Multilayered silicone foam dressing was used for the axilla and greater trochanter.

**Figure 3:**
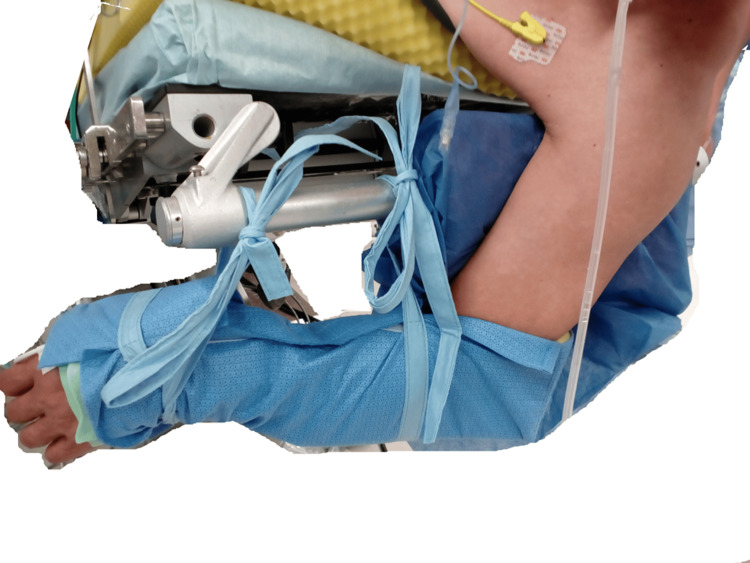
Fixing the lower upper limb The lower upper limb was fixed in a suspended position.

A Mayfield Head Three-Point Fixator® 4-0-A1059 (Ohwa Tsusho Co., Ltd., Japan) was used as the head. Multitask Armboard Ver. 2® 08-086-02 (Mizuho Corporation, Tokyo, Japan) and Parkbench RT-01® (Hopes Co., Ltd., Japan) were used for upper and lower limb fixation, respectively. The pubic, sacral, and sternal regions were fixed in the operative position with a multi-lateral support 300 mm type Ver. 2 (Mizuho Corporation). The operating table and mattresses used were the MST-7201 BX microsurgery operating table® (Mizuho Corporation), MPO mattress® (Mizuho Corporation), and Charming mat plus single® (Mizuho Corporation); axillary pillows were not used. For the fibula, Bonmat no. 2® (ALCARE Co., Ltd., Japan) was used. Body pressure was measured on the lower side of the chest using SR Soft Vision® (numerical version) (Sumitomo Riko Co. Ltd., Japan) (Figures 1-3). The 3M™ Bair Hugger™ patient warming unit, model 675 (3M, St. Paul, MN, USA) was used for intraoperative temperature control. The intraoperative central temperature was measured using the esophageal temperature.

Control Group

**Figure 4:**
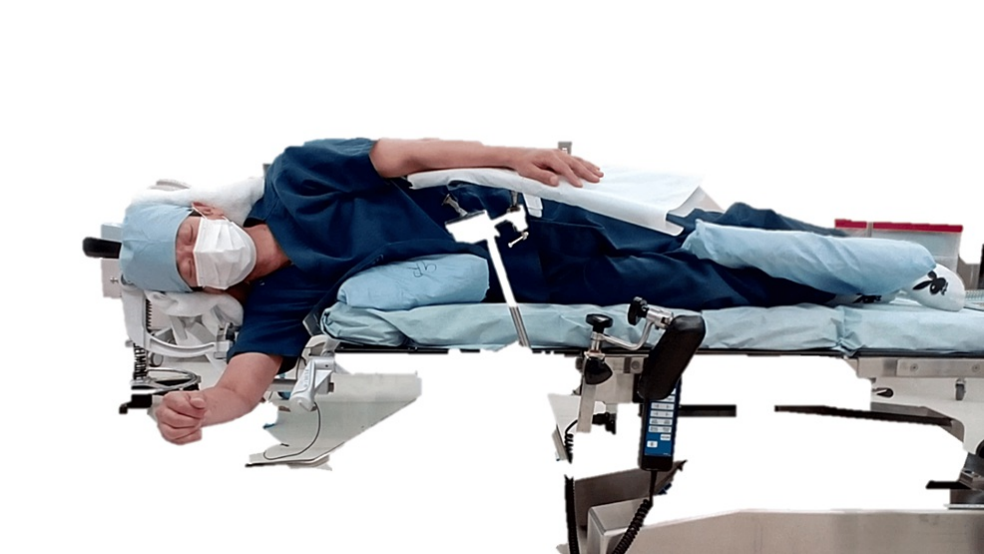
Overview of postural fixation in the control group Only the mattress attached to the operating table was used, an axillary pillow was inserted, and both upper limbs were fixed on the upper table.

A control group was included in the study between January 2017 and June 2019. After admission to the operating room, the ESENTA Skin Capsule Spray® (#423288; ConvaTec) was applied as a skin protectant to the lateral chest, greater trochanter, fibula head, and malleolus in the lateral decubitus position. Permirol® H24R05 (Nitto Denko Corporation, Japan) was then applied to the lateral chest, greater trochanter, fibular head, and malleolus. A Mayfield Head Three-Point Fixator® 4-0-A1059 (Ohwa Tsusho Co., Ltd.), Multitask Armboard Ver. 2® 08-086-02 (Mizuho Corporation), Softnurse® 20923 (ALCARE Co., Ltd.), and Bonmat no. 2® (ALCARE Co., Ltd.) were used for the fixation of the head, upper limb, axilla, and fibula, respectively. The 3M™ Bair Hugger™ patient warming unit, model 675 (3M) was used for intraoperative temperature control. The intraoperative central temperature was measured using the esophageal temperature (Figure 4).

Statistical analysis

Patient background and surgical and anesthetic factors are shown as medians (interquartile ranges). Differences in the primary and secondary endpoints, patient characteristics, and surgical and anesthetic factors between the intervention and control groups were compared. Two-tailed p-values were used, and a difference of <0.05 was considered statistically significant. All statistical analyses were performed using JMP® 16 software (SAS Institute Inc., Cary, NC, USA). The Mann-Whitney U test was used for group comparisons of continuous variables. The chi-squared test or Fisher's exact test was used for nominal variables.

## Results

Participant demographics

**Figure 5:**
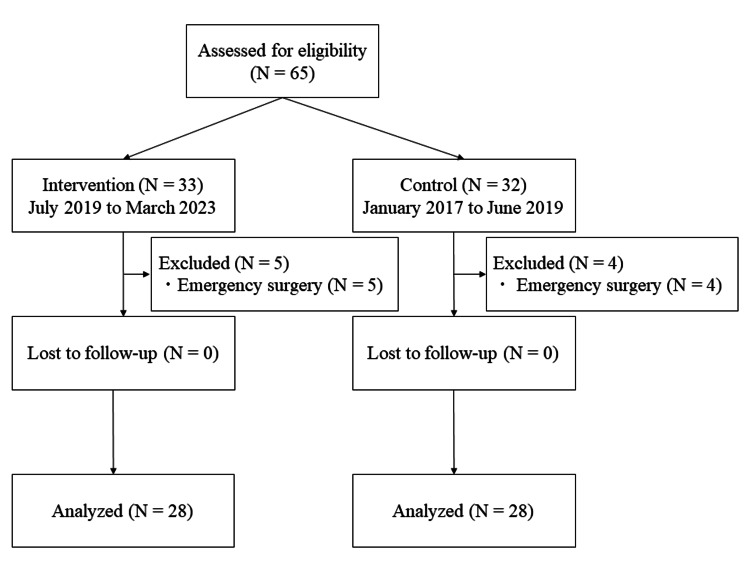
Trial STROBE flowchart During the study period, 65 patients were included, and 56 patients were analyzed after excluding emergency surgery cases. STROBE, Strengthening the Reporting of Observational Studies in Epidemiology

During the study period, 65 patients were included, and 56 patients (28 in the intervention group and 28 in the control group) were analyzed after excluding emergency surgery cases (Figure 5). The patient characteristics are shown in Table 1. There were no significant differences in patient characteristics or surgical or anesthetic factors between the two groups.

**Table 1:** Patient characteristics Values are presented as medians (ranges) or number of patients (%). The Mann-Whitney U test was used for comparisons of ordinal data. Nominal data were compared using the chi-squared test or Fisher’s exact test.

	Intervention group (n=28)	Control group (n=28)	p value
Age (years)	63.0 (50.3-75.3)	57.0 (51.3-73.8)	.59
Sex			.99
Male	11 (39.3%)	11 (39.3%)	
Female	17 (60.7%)	17 (60.7%)	
Height (cm)	157.2 (152.5-163.7)	159.0 (151.0-166.6)	.67
Weight (kg)	59.5 (50.1-68.0)	55.8 (48.6-66.1)	.15
Body mass index (kg/m^2^)	23.4 (22.6-27.4)	23.3 (21.1-23.8)	.06
Preoperative total protein (g/dl)	6.5 (6.0-7.0)	6.6 (6.0-7.0)	.84
Preoperative albumin (g/dl)	4.2 (3.8-4.4)	4.0 (3.7-4.4)	.46
Preoperative hemoglobin (g/dl)	14.2 (12.3-15.1)	13.1 (12.0-14.0)	.20
Paralysis			.99
Yes	0 (0%)	1 (3.6%)	
No	28 (100%)	27 (96.4%)	
Edema			.97
Yes	1 (3.6%)	2 (7.2%)	
No	27 (96.4%)	26 (92.8%)	

The operative and anesthetic factors are listed in Table 2. The mean intraoperative body pressure on the axilla was significantly lower in the intervention group (p < .01).

**Table 2:** Postoperative factors Values are presented as number of patients (%). Nominal data were compared using Fisher’s exact test. *Significant difference between groups.

	Intervention group (n=28)	Control group (n=28)	p value
Postoperative pressure injury (axilla and greater trochanter)			< .01*
Yes	0 (0%)	17 (60.7%)	
Reactive hyperemia		12 (70.6%)	
Stage Ⅰ		4 (23.5%)	
Stage Ⅱ		1 (5.9%)	
No	28 (100%)	11 (39.3%)	
Postoperative pressure injury (except for axilla and greater trochanter)			.66
Yes	2 (7.2%)	4 (14.4%)	
Reactive hyperemia	2 (100%)	3 (75.0%)	
Ankle	2 (100%)	2 (66.7%)	
Upper limbs		1 (33.3%)	
Stage Ⅰ	0 (0%)	1 (25.0%)	
Upper limbs		1 (100%)	
No	26 (92.8%)	24 (85.6%)	
Postoperative neurological disorders			.99
Yes	0 (0%)	1 (3.6%)	
No	28 (100%)	27 (96.4%)	

The postoperative factors are listed in Table 3. There were 17 cases of postoperative pressure injury and skin disorders in the axilla and greater trochanter in the control group but none in the intervention group (p < .01). Postoperative pressure injury and skin disorders other than those of the axilla and greater trochanter occurred in two patients in the intervention group and four cases in the control group (p = .66). There were no postoperative neurological disorders in the intervention or control group (p = .99).

**Table 3:** Surgical and anesthetic factors Values are presented as medians (ranges). The Mann-Whitney U test was used for comparisons of ordinal data. *Significant difference between groups.

	Intervention group (n=28)	Control group (n=28)	p value
Operative time (min)	253.0 (227.0-314.5)	272 (207.5-354.3)	.66
Anesthesia time (min)	390.0 (337.3-408.8)	357.5 (295.3-511.3)	.82
Amount of bleeding (ml)	137.5 (68.8-205)	127.5 (62.5-198.5)	.70
Total fluid volume (ml)	2571.5 (2248.8-2836.3)	2280.0 (2015.0-2682.5)	.32
Urine volume (ml)	745.0 (500.0-920.0)	776 (548.8-1256.3)	.06
Intraoperative mean body pressure (mmHg)	40 (35-44)	55.5 (42.3-69.5)	< .01*
Intraoperative mean central temperature (°C)	36.7 (36.2.-37.0)	36.6 (36.3.-37.1)	.97

## Discussion

We were able to obtain a significant reduction in the occurrence of pressure ulcers and skin disorders after the neurosurgery park bench position surgery in patients, which is a high-risk group for pressure injury. The factors that enabled us to obtain this effect were analyzed.

First, an important countermeasure against pressure injury in the perioperative period is body pressure intervention. It is important to distribute body pressure by expanding the contact area between the body and mattress when the patient is in an operative position. Currently, devising pressure redistribution and postural fixation using a pressure-dispersing mattress is an essential countermeasure for perioperative pressure ulcers [20,21]. The body pressure on the axilla in the intervention group was significantly lower than that in the control group. A pressure-dispersing mattress was used in the intervention group in addition to the mattress attached to the operating table. This made it possible to extend contact with the body of the subject. In addition to the effect of the additional pressure-dispersing mattress, the removal of the axillary pillow may have reduced the body pressure on the axilla and lateral chest, leading to a reduction in the risk of pressure injury. This was made possible by monitoring the intraoperative body pressure in the axillary region. Monitoring body pressure has also been reported as an effective means to ensure and complement measures to prevent pressure ulcers [22]. Body pressure monitoring can be used as an objective index for the assessment of intraoperative interventions. Since it is impossible to change the position of the patient during the operation, it is necessary to perform decompression during the operation to ensure that there is no hindrance to the operation, to prevent an increase in body pressure. In this study, only monitoring the body pressure of the axillary region was performed; however, there is a need to monitor the body pressure of the greater trochanter. In the control group, postoperative pressure injuries, skin disorders of the lower upper extremities, and neurological disorders were observed. In the intervention group, there were no pressure injuries, skin disorders, or neurological disorders because the method was changed to the suspended fixation method. Suspension of the lower upper limb is believed to eliminate local pressure and disperse pressure.

Second, it is necessary to adjust the position of the patient so that the operation can be performed safely, such as by rotating the body of the patient to the head or foot, inserting a pillow, and performing the operating table rotation during the operation. Shear forces can occur when the position is adjusted. Therefore, slippage must be reduced through immobilization and skin protection. The dressing method utilized can create a 'sliding effect' [23]. In the conventional method, polyurethane foam dressing is applied to the axilla and greater trochanter, the most common sites of pressure ulcers in the lateral position. These dressings have also been reported to prevent shear and friction [24]. However, previous studies have reported increased preventive effects of multilayered silicone foam dressings on pressure ulcer development as compared to polyurethane foam dressings [18,25]. In the intervention group in this study, the multilayered silicone foam dressing was more effective in preventing slippage and friction caused by surgical position fixation, which may have contributed to the decrease in the incidence of pressure ulcers after surgery.

Third, the microclimate is a critical factor. The microclimate has been proposed to be one of the causes of pressure ulcers (in 2010) and is defined as the temperature of the skin surface or tissue and the wetness of the body and skin surface [26]. In the human body, an elevated body temperature is associated with increased tissue metabolism [27]. Increased metabolism heightens oxygen and nutrient consumption, subsequently elevating the body's metabolic demands. In addition, the administration of muscle relaxants may lead to vascular occlusion by reducing muscle elasticity and could lead to ischemia. These ischemic conditions tend to lack oxygen and nutrient requirements under high temperature and humidity conditions. Therefore, high temperatures and humidity can easily lead to tissue damage. In this study, the median intraoperative central temperature was within the 36°C range in both the intervention and control groups, which is within the range of the central temperature in the human body (36.8-37.0°C), and unplanned periodic hyperthermia is defined as a decrease in the central temperature below 36°C [28]. At the central temperature, no significant difference was observed, but the intervention group using multilayered silicone foam dressings might have been able to maintain adequate humidity. Multilayered silicone foam dressings have been reported to have regulatory functions in wet environments [29,30]. Therefore, the constructive management of microclimates by the application of multilayered silicone foam dressings in the intervention group may have contributed to the prevention of postoperative pressure injury and skin disorders.

In this study, we were able to significantly reduce the incidence of postoperative pressure injury and skin disorders in neurosurgery park bench position surgery. It is essential to implement management strategies for body pressure, slippage, friction, and microclimate, seen here as part of the revised measures in the intervention group. However, one of the limitations and challenges of this study is the small amount of data analyzed, because the study was conducted in a single institutional setting involving a single cohort of participants. We believe that further data collection and statistical analysis are necessary in the future. In addition, although this study was intended for elective surgery under general anesthesia, we believe it is necessary to conduct further analysis in patients undergoing emergency surgery, those with poor nutritional status, and those undergoing prolonged surgery.

## Conclusions

Our study rigorously examined the impact of proactive measures to mitigate postoperative pressure injuries and skin disorders in patients undergoing neurosurgical procedures in the park bench position. The results unequivocally demonstrated a significant reduction in the incidence of these complications within the intervention group, which received comprehensive management of body pressure, temperature, humidity, and microclimate compared to the control group. The intervention's core comprised the utilization of pressure-dispersing mattresses and multilayered silicone foam dressings, aimed at optimizing the surgical bed's microenvironment. This approach not only ensured the even distribution of body pressure but also effectively managed the microclimate around high-risk areas for pressure injuries. Such meticulous attention to detail in managing these variables underscored the effectiveness of our preventative measures. A comparative analysis between the intervention and control groups highlights the substantial benefits of our approach. The intervention group exhibited no cases of postoperative pressure injuries or skin disorders, a testament to the efficacy of our preventive strategies. These findings are not only statistically significant but also clinically relevant, indicating a clear pathway toward enhancing patient care in neurosurgical practices. The implications of our study are far-reaching, suggesting a paradigm shift in how perioperative care is approached for patients in vulnerable positions. The adoption of our preventative measures could standardize care protocols, significantly reducing the risk of pressure injuries and enhancing overall patient outcomes in neurosurgical settings. Future research should aim to explore the applicability of these interventions across a broader spectrum of surgical positions and durations. Additionally, further studies are needed to refine these strategies and validate their effectiveness in diverse clinical settings. Despite the study's limitations, including its focus on a single surgical position and setting, its strengths lie in its prospective design and the clear delineation of intervention impacts. These factors contribute to the robustness of our conclusions and the potential for widespread clinical application.

In conclusion, our study provides compelling evidence for the adoption of comprehensive preventative measures against postoperative pressure injuries and skin disorders in neurosurgery. By integrating body pressure, temperature, humidity, and microclimate management into standard care protocols, we can significantly enhance patient care and outcomes in the neurosurgical field.

## References

[1] National Pressure Ulcer Advisory Panel, European Pressure Ulcer Advisory Panel, Pan Pacific Pressure Injury Alliance (2014). Prevention and Treatment of Pressure Ulcers: Quick Reference Guide. https://web.archive.org/web/20180424024843id_/http://www.npuap.org/wp-content/uploads/2014/08/Quick-Reference-Guide-DIGITAL-NPUAP-EPUAP-PPPIA-Jan2016.pdf.

[2] Karahan E, Ayri AU, Çelik S (2022). Evaluation of pressure ulcer risk and development in operating rooms. J Tissue Viability.

[3] Latimer S, Chaboyer W, Gillespie B (2014). Patient participation in pressure injury prevention: giving patient's a voice. Scand J Caring Sci.

[4] Gaspar S, Peralta M, Marques A, Budri A, Gaspar de Matos M (2019). Effectiveness on hospital-acquired pressure ulcers prevention: a systematic review. Int Wound J.

[5] Riemenschneider KJ (2018). Prevention of pressure injuries in the operating room: a quality improvement project. J Wound Ostomy Continence Nurs.

[6] Teleten O, Prevatt J, Peterson L, Burleson C, Wilson M, Kirkland-Kyhn H (2021). Use of pressure mapping to compare two operating room surfaces in the supine with bent knees position and the supine in lithotomy position. Wounds.

[7] Kirkland-Walsh H, Teleten O, Wilson M, Raingruber B (2015). Pressure mapping comparison of four OR surfaces. AORN J.

[8] Kitamura A, Yoshimura M, Nakagami G, Yabunaka K, Sanada H (2019). Changes of tissue images visualised by ultrasonography in the process of pressure ulcer occurrence. J Wound Care.

[9] Marotta DA, Brazdzionis J, Fiani B, Duong J, Noel J, Siddiqi J (2021). Perioperative positioning in neurosurgery: a technical note on park bench positioning for the obese patient using the "arrowhead" technique. Cureus.

[10] Yoshimura M, Iizaka S, Kohno M (2016). Risk factors associated with intraoperatively acquired pressure ulcers in the park-bench position: a retrospective study. Int Wound J.

[11] Yoshimura M, Nakagami G, Iizaka S (2015). Microclimate is an independent risk factor for the development of intraoperatively acquired pressure ulcers in the park-bench position: a prospective observational study. Wound Repair Regen.

[12] Burlingame BL (2017). Guideline implementation: positioning the patient. AORN J.

[13] Mervis JS, Phillips TJ (2019). Pressure ulcers: prevention and management. J Am Acad Dermatol.

[14] Spruce L (2017). Back to basics: preventing perioperative pressure injuries. AORN J.

[15] Cheng A, Kessler D, Mackinnon R (2016). Reporting guidelines for health care simulation research: extensions to the CONSORT and STROBE statements. Adv Simul (Lond).

[16] Kharasch ED (2019). Observations and observational research. Anesthesiology.

[17] Eberhardt TD, de Lima SB, de Avila Soares RS (2021). Prevention of pressure injury in the operating room: heels operating room pressure injury trial. Int Wound J.

[18] Yoshimura M, Ohura N, Tanaka J (2018). Soft silicone foam dressing is more effective than polyurethane film dressing for preventing intraoperatively acquired pressure ulcers in spinal surgery patients: the Border Operating room Spinal Surgery (BOSS) trial in Japan. Int Wound J.

[19] Edsberg LE, Black JM, Goldberg M, McNichol L, Moore L, Sieggreen M (2016). Revised National Pressure Ulcer Advisory Panel Pressure Injury Staging System: Revised Pressure Injury Staging System. J Wound Ostomy Continence Nurs.

[20] Mamom J, Daovisan H (2022). Repositioning mattress: how a lateral tilt position reshapes the prevention of pressure ulcers in bedridden patients. J Med Eng Technol.

[21] Defloor T, De Schuijmer JD (2000). Preventing pressure ulcers: an evaluation of four operating-table mattresses. Appl Nurs Res.

[22] Mansfield S, Obraczka K, Roy S (2020). Pressure injury prevention: a survey. IEEE Rev Biomed Eng.

[23] Call E, Pedersen J, Bill B (2015). Enhancing pressure ulcer prevention using wound dressings: what are the modes of action?. Int Wound J.

[24] Moore ZE, Webster J (2018). Dressings and topical agents for preventing pressure ulcers. Cochrane Database Syst Rev.

[25] Forni C, Gazineo D, Allegrini E (2022). Effectiveness of a multi-layer silicone-adhesive polyurethane foam dressing as prevention for sacral pressure ulcers in at-risk in-patients: randomized controlled trial. Int J Nurs Stud.

[26] Wounds International (2010). International Review. Pressure Ulcer Prevention: Pressure, Shear, Friction and Microclimate in Context. A Consensus Document. https://woundsinternational.com/wp-content/uploads/sites/8/2023/02/5a517b64dacfb4fee06c221412f0b4e9.pdf.

[27] Zaretsky DV, Romanovsky AA, Zaretskaia MV, Molkov YI (2018). Tissue oxidative metabolism can increase the difference between local temperature and arterial blood temperature by up to 1.3°C: implications for brain, brown adipose tissue, and muscle physiology. Temperature (Austin).

[28] Forbes SS, Eskicioglu C, Nathens AB, Fenech DS, Laflamme C, McLean RF, McLeod RS (2009). Evidence-based guidelines for prevention of perioperative hypothermia. J Am Coll Surg.

[29] Schwartz D, Levy A, Gefen A (2018). A computer modeling study to assess the durability of prophylactic dressings subjected to moisture in biomechanical pressure injury prevention. Ostomy Wound Manage.

[30] Gefen A, Alves P, Creehan S, Call E, Santamaria N (2019). Computer modeling of prophylactic dressings: an indispensable guide for healthcare professionals. Adv Skin Wound Care.

